# Supplementary material for: Effects of Post-Harvest Elicitor Treatments with Ultrasound, UV- and Photosynthetic Active Radiation on Polyphenols, Glucosinolates and Antioxidant Activity in a Waste Fraction of White Cabbage (Brassica oleracea var. capitata)
Source: Molecules. 2022 Aug 17;27(16):5256. doi: 10.3390/molecules27165256 (PMC9414070; doi:10.3390/molecules27165256)
Supplement: Supplementary file 1 [file molecules-27-05256-s001.zip › molecules-1831127-supplementary.pdf]

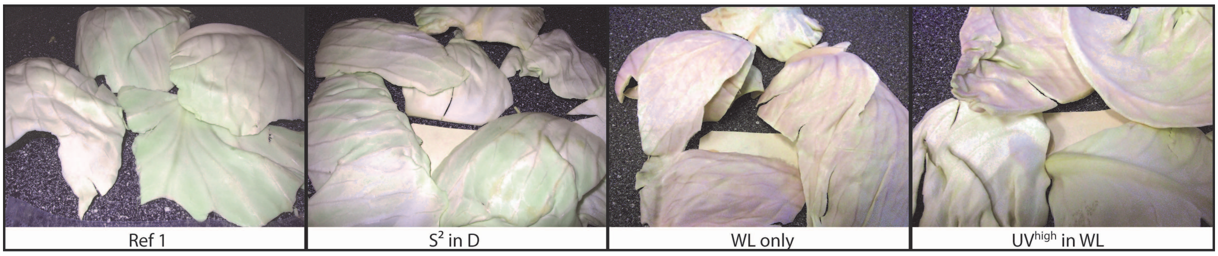


Figure S1. Sample of photos of cabbage leaves after final radiation exposure and 5 h in darkness before sampling to analyses.
